# Sexual function and satisfaction among heterosexual and sexual minority U.S. adults: A cross-sectional survey

**DOI:** 10.1371/journal.pone.0174981

**Published:** 2017-04-12

**Authors:** Kathryn E. Flynn, Li Lin, Kevin P. Weinfurt

**Affiliations:** 1 Center for Patient Care and Outcomes Research, Medical College of Wisconsin, Milwaukee, Wisconsin, United States of America; 2 Duke Clinical Research Institute, Duke University School of Medicine, Durham, North Carolina, United States of America; 3 Department of Psychiatry and Behavioral Sciences Duke University School of Medicine, Durham, North Carolina, United States of America; University of Westminster, UNITED KINGDOM

## Abstract

**Background:**

Despite known health disparities for sexual minorities, few studies have described sexual function by sexual orientation using a robust approach to measurement of sexual function. We compared recent sexual function and satisfaction by sexual orientation among English-speaking US adults.

**Methods and findings:**

Cross-sectional surveys were administered by KnowledgePanel^®^ (GfK), an online panel that uses address-based probability sampling and is representative of the civilian, noninstitutionalized US population. Data were collected in 2013 from the general population (n = 3314, 35% response rate) and in 2014 from self-identified lesbian, gay, and bisexual adults (n = 1011, 50% response rate). Sexual function and satisfaction were measured using the Patient-Reported Outcomes Measurement Information System^®^ Sexual Function and Satisfaction measure version 2.0 (PROMIS SexFS v2). The PROMIS SexFS v2 is a comprehensive, customizable measurement system with evidence for validity in diverse populations. A score of 50 (SD 10) on each domain corresponds to the average for US adults sexually active in the past 30 days. We adjusted all statistics for the complex sample designs and report differences within each sex where the 95% CIs do not overlap, corresponding to p<0.01. Among US men who reported any sexual activity in the past 30 days, there were no differences in erectile function or orgasm-ability. Compared to heterosexual men, sexual minority men reported higher oral dryness and lower orgasm-pleasure and satisfaction. Compared to heterosexual men, gay men reported lower interest, higher anal discomfort and higher oral discomfort. Among sexually active women, there were no differences in the domains of vulvar discomfort-clitoral, orgasm-pleasure, or satisfaction. Compared to heterosexual women, sexual minority women reported higher oral dryness. Lesbian women reported lower vaginal discomfort than other women; lesbian women reported higher lubrication and orgasm-ability than heterosexual women. Bisexual women reported higher interest, higher vulvar discomfort-labial and higher anal discomfort than other women, as well as higher oral discomfort compared to heterosexual women.

**Conclusions:**

Recent sexual function and satisfaction differed by sexual orientation among US adults. Sexual minority men and women had decrements in domains of sexual function that have not traditionally been included in multi-dimensional self-report measures. Clinicians should make themselves aware of their patients’ sexual concerns and recognize that sexual minority patients may be more vulnerable to certain sexual difficulties than heterosexual patients.

## Introduction

Health disparities by sexual orientation are well-documented [1]. Most research comparing heterosexual and sexual minority adults has focused on mental health, substance use, and sexually transmitted diseases, including HIV. Fewer studies have examined differences in sexual function and satisfaction, though these are important components of quality of life for many people [2]. A study of the health of adults aged 18–65 years attending general practice clinics in London, UK [3] and a population-based study of adults aged 19–70 years from the Netherlands [4] found similar numbers of reported sexual problems/dysfunctions regardless of sexual orientation, while two US studies have suggested there may be differences in particular domains. For example, in a convenience sample of men aged 18–81 years, more erectile difficulties were reported by gay men compared to heterosexual men [5]. Among women, higher desire and more sexual satisfaction were reported by lesbian women compared to heterosexual women in a convenience sample of women aged 19–62 years [6]. However no large, US-based study has examined sexual function and satisfaction by sexual orientation in a probability-based sample.

Our objective was to provide epidemiologic data on recent sexual function and satisfaction among English-speaking US heterosexual, gay, lesbian, and bisexual adults. We used the PROMIS Sexual Function and Satisfaction (SexFS) measure version 2.0, which has evidence for validity in diverse target populations, including both heterosexual and sexual minority men and women of all ages and literacy levels. The PROMIS SexFS covers a wide set of domains, including many not typically assessed in older measures. We compared PROMIS SexFS scores by sex and sexual orientation.

## Methods

The Patient-Reported Outcomes Measurement Information System^®^ (PROMIS^®^) was created by the National Institutes of Health to improve the measurement of patient-reported health. PROMIS instrument development and validation standards emphasize both qualitative development of measures to ensure comprehensive coverage of concepts and to improve comprehension of items, including by those with low literacy [7], as well as state-of-the-science psychometric evaluation of measures based on item response theory in addition to classical test theory [8].

The PROMIS SexFS v2 is a comprehensive, customizable measurement system with evidence for validity in diverse populations [9–13]. We used two screener questions from the measure to construct a variable for recent sexual activity status with three categories. The first question asked, “In the past 30 days, did you have any type of sexual activity? (Examples of sexual activity are masturbation, oral sex, and sexual intercourse.)” If a “No” response was provided, we categorized as “no sexual activity”. If “Yes”, a second question asked, “In the past 30 days, did you have any type of sexual activity with a partner? (Examples of sexual activity with a partner are oral sex and sexual intercourse.)” If a “No” response was provided to this question, we categorized as “sexual activity—alone” and if “Yes”, we categorized as “sexual activity—with a partner (and alone, if applicable)” The PROMIS SexFS v2 includes 17 domains total, 12 of which are reported in this study. The PROMIS SexFS v2 uses a 30-day recall period, and apart from the screener questions, each question uses a 5-level polytomous response set, e.g., “In the past 30 days, how often have you felt like you wanted to have sexual activity? Never, rarely, sometimes, often, always.” PROMIS scores are standardized and centered on the US adult population; and for the domains within the PROMIS SexFS v2, a score of 50 (SD 10) on each domain corresponds to the average for US adults sexually active in the past 30 days.

Cross-sectional surveys were administered by KnowledgePanel^®^ (GfK), an online panel that uses address-based probability sampling and is representative of the civilian, noninstitutionalized US population [14]. Invitations were sent and data was collected in June 2013 from the general population and again in July 2014 to panel members who had indicated they are gay, lesbian, or bisexual in order to increase the available sample of self-identified sexual minorities (“Do you consider yourself to be… heterosexual or straight, gay, lesbian, bisexual, other.”). There was no explicit refusal conversion process; instead, enrollment ended when the targeted sample size was met, 3500 for the general population based on pre-planned psychometric analyses for measure development and 1000 for the sexual minority sample based on available funding. Eligibility criteria for both samples included age of 18 years or older and ability to read English.

Because the same sexual minorities may have participated in both surveys, we removed the 164 sexual minorities from the general population survey for analysis. Analyses were weighted for inferences to the national population and accounted for the complex sample design. Since the two samples had independent weights and thus could not be compared directly, we estimated means with 95% CIs and used these to make group comparisons. We report differences within each sex where the CIs do not overlap, corresponding to p<0.01 [15]. The institutional review boards of the Duke University Health System and the Medical College of Wisconsin approved the studies; participants provided online consent.

## Results

In the general population 42% (4292/10129) responded to the survey and 35% (3516/10129) were eligible and participated in the survey; for the sexual minority sample 55% (1114/2016) responded and 50% (1011/2016) were eligible and participated. Compared to non-respondents, respondents in both samples were older and more often white and non-Hispanic (Table 1). Respondents in the sexual minority sample were also more often male, with higher education, lower annual household income, and less often from the South (US geographic region).

**Table 1:** The sociodemographics of survey respondents and non-respondents compared to the current population survey.

Characteristic	General Population Sample			Sexual Minority Sample			Current Population Survey^c^
	Respondents^a^	Nonrespondents	Respondent Weighted^b^	Respondents^a^	Nonrespondents	Respondent Weighted	
	(n = 4292)	(n = 5,837)	(n = 3314)	(n = 1,114)	(n = 902)	(n = 1011)	(n = 242,248)
Age (years), mean	50	44	47	48	40	41	47
Age (years), No. (%)							
18 to 29	670 (15.6%)	1,634 (28.0%)	698 (21.1%)	223 (20.0%)	318 (35.3%)	342 (33.9%)	51,983 (21.5%)
30 to 44	996 (23.2%)	1,652 (28.3%)	827 (24.9%)	236 (21.2%)	260 (28.8%)	266 (26.4%)	61,058 (25.2%)
45 to 59	1,322 (30.8%)	1,436 (24.6%)	923 (27.8%)	362 (32.5%)	208 (23.1%)	284 (28.1%)	64,215 (26.5%)
≥ 60	1,305 (30.4%)	1,115 (19.1%)	866 (26.1%)	293 (26.3%)	116 (12.9%)	118 (11.7%)	64,992 (26.8%)
Female Sex	2,219 (51.7%)	3,035 (52.0%)	1652 (49.9%)	473 (46.8%)	548 (60.8%)	536 (53.0%)	125,197 (51.7%)
Race							
White	3,532 (82.3%)	4,243 (72.7%)	2632 (79.4%)	938 (84.2%)	670 (74.3%)	797 (78.9%)	190,616 (78.7%)
Black/African American	451 (10.5%)	981 (16.8%)	403 (12.2%)	78 (7.0%)	124 (13.7%)	118 (11.7%)	30,097 (12.4%)
Asian	94 (2.2%)	175 (3.0%	37 (1.1%)	21 (1.9%)	29 (3.2%)	22 (2.2%)	14,041 (5.8%)
American Indian or Alaska Native	26 (0.6%)	58 (1.0%)	131 (4.0%)	8 (0.7%)	8 (0.9%)	6 (0.6%)	2,632 (1.1%)
Native Hawaiian/Pacific Islander	13 (0.3%)	35 (0.6%)	13 (0.4%)	4 (0.4%)	1 (0.1%)	6 (0.6%)	969 (0.4%)
2 or more races	176 (4.1%)	350 (6.0%)	98 (3.0%)	65 (5.8%)	70 (7.8%)	62 (6.1%)	3,893 (1.6%)
Hispanic Ethnicity	408 (9.5%)	957 (16.4%)	429 (13.0%)	138 (12.4%)	163 (18.1%)	134 (13.3%)	37,509 (15.5%)
Education level							
Less than high school	395 (9.2%)	747 (12.8%)	372 (11.2%)	32 (2.9%)	48 (5.3%)	82 (8.1%)	29,659 (12.2%)
High school	1,356 (31.6%)	1,646 (28.2%)	998 (30.1%)	118 (10.6%)	132 (14.6%)	209 (20.7%)	71,654 (29.6%)
Some college	1,288 (30.0%)	1,885 (32.3%	966 (29.2%)	405 (36.4%)	387 (42.9%)	354 (35.0%)	68,749 (28.4%)
Bachelor's degree or higher	1,253 (29.2%)	1,558 (26.7%)	977 (29.5%)	559 (50.2%)	335 (37.1%)	366 (36.2%)	72,186 (29.8%)
Annual household income 2014							
< $50,000	1,876 (43.7%)	2,755 (47.2%)	1376 (41.5%)	574 (51.5%)	505 (56.0%)	524 (51.8%)	122,579 (38.8%)
$50,000-$99,999	1,395 (32.5%)	1,780 (30.5%)	1155 (34.9%)	340 (30.5%)	262 (29.0%)	301 (29.8%)	96,972 (30.7%)
≥ $100,000	1,017 (23.7%)	1,302 (22.3%)	783 (23.6%)	200 (18.0%)	135 (15.0%)	186 (18.4%)	96,616 (30.6%)
Region							
Northeast	755 (17.6%)	1,027 (17.6%)	587 (17.7%)	185 (16.6%)	160 (17.7%)	199 (19.7%)	43,767 (18.1%)
Midwest	1,052 (24.5%)	1,220 (20.9%)	738 (22.3%)	247 (22.2%)	197 (21.8%)	168 (16.6%)	51,528 (21.3%)
South	1,532 (35.7%)	2,241 (38.4%)	1226 (37.0%)	352 (31.6%)	290 (32.2%)	356 (35.2%)	90,130 (37.2%)
West	953 (22.2%)	1,348 (23.1%)	762 (23.0%)	330 (29.6%)	255 (28.3%)	288 (28.4%)	56,823 (23.5%)

^a^. Includes those who responded to the survey invitation including those who were then screened out.

^b^. Excludes those who identified as sexual minority, as the same sexual minority individuals could have participated in both surveys.

^c^. National data generated from the CPS ASEC (Annual Social and Economic Supplement) 2015 dataset. http://www.census.gov/cps/data/cpstablecreator.html.

Heterosexual men and women were more often married than sexual minority men and women (Table 2). Gay men more often reported being in a civil union or domestic partnership or living with a partner compared to other men, as did lesbian women compared to heterosexual women. Compared to heterosexual men, gay and bisexual men were more often single and not dating; this difference was not seen among women. Heterosexuals were more often sexually active with a partner in the past 30 days compared to sexual minorities, whereas sexual minorities were more often sexually active without a partner compared to heterosexuals. A smaller proportion of bisexual women reported no sexual activity in the past 30 days compared to other women.

**Table 2:** Current relationship status and sexual activity in past 30 days by self-reported sex and sexual orientation.

	Men			Women		
	Heterosexual	Gay or Lesbian	Bisexual	Heterosexual	Gay orLesbian	Bisexual
	(n = 1662)	(n = 334)	(n = 141)	(n = 1652)	(n = 199)	(n = 337)
Current relationship status, % (95% CI)						
Married	57.5 (54.6, 60.5)	8.4 (5.5, 11.4)	34.7 (24.7, 44.7)	51.1 (47.8, 54.4)	21.9 (15.7, 28.2)	31.1 (24.9, 37.3)
In a civil union or domestic partnership	0.9 (0.3, 1.6)	5.2 (2.6, 7.8)	0.9 (0.0, 2.1)	0.4 (0.1, 0.7)	8.1 (3.9, 12.3)	2.7 (0.0, 5.5)
Living with a partner	7.9 (6.2, 9.6)	26.7 (20.3, 33.0)	7.2 (2.0, 12.4)	9.4 (7.3, 11.5)	27.1 (19.4, 34.7)	16.3 (11.2, 21.4)
In a relationship but not living together	6.6 (5.0, 8.1)	12.2 (7.4, 17.0)	11.5 (2.9, 20.1)	7.7 (5.9, 9.6)	14.1 (8.3, 19.9)	15.3 (9.9, 20.7)
Single but dating 1 or more people	4.5 (3.3, 5.8)	5.3 (2.9, 7.7)	2.1 (0.0, 4.2)	4.8 (3.3, 6.4)	7.3 (2.8, 11.7)	6.8 (3.2, 10.3)
Single and not dating	20.8 (18.4, 23.3)	42.0 (35.6, 48.4)	42.2 (30.7, 53.7)	21.8 (18.9, 24.6)	20.7 (14.5, 26.9)	24.7 (18.4, 31.0)
Other	1.7 (1.0, 2.4)	0.2 (0.0, 0.5)	1.3 (0.0, 2.7)	4.8 (3.4, 6.2)	0.9 (0.0, 1.9)	3.2 (0.0, 6.5)
Sexual activity status in past 30 days, % (95% CI)						
No sexual activity	16.1 (14.0, 18.3)	11.9 (7.3, 16.4)	11.0 (3.2, 18.7)	31.7 (28.6, 34.8)	28.5 (21.4, 35.6)	13.8 (9.3, 18.3)
Sexual activity—alone	15.4 (13.3, 17.5)	31.7 (25.4, 37.9)	40.5 (29.0, 51.9)	12.0 (9.8, 14.2)	24.7 (17.5, 31.8)	20.6 (15.0, 26.3)
Sexual activity—with a partner (and alone, if applicable)	68.5 (65.8, 71.2)	56.5 (49.8, 63.1)	48.6 (37.3, 59.9)	56.3 (53.0, 59.6)	46.8 (38.8, 54.9)	65.6 (59.0, 72.2)

Among men who reported any sexual activity (with or without a partner) in the past 30 days, there were no differences by sexual orientation in either erectile function or orgasm-ability (Table 3). Compared to heterosexual men, sexual minority men reported higher oral dryness (4–5 points) and lower orgasm-pleasure (2–4 points) and satisfaction (4–5 points). Compared to heterosexual men, gay men reported lower interest (3 points), higher anal discomfort (9 points), and higher oral discomfort (5 points). Among sexually active women (with or without a partner), there were no differences in the domains of vulvar discomfort-clitoral, orgasm-pleasure, or satisfaction. Compared to heterosexual women, sexual minority women reported higher oral dryness (5–7 points). Lesbian women reported lower vaginal discomfort (4 points) than other women; lesbian women reported higher lubrication (4 points) and orgasm-ability (4 points) than heterosexual women. Bisexual women reported higher interest (3 points), higher vulvar discomfort-labial (2–3 points) and higher anal discomfort (3–4 points) than other women, as well as higher oral discomfort (5 points) compared to heterosexual women.

**Table 3:** Sexual function and satisfaction in past 30 days of sexually active men and women by self-reported sexual orientation.

PROMIS SexFS v2 Domains (maximum # items)	Men			Women		
	Heterosexual	Gay or Lesbian	Bisexual	Heterosexual	Gay or Lesbian	Bisexual
	(n = 1382)	(n = 293)	(n = 121)	(n = 1118)	(n = 141)	(n = 289)
	PROMIS SexFS Scores^a^, mean T-score (95% CI)					
Interest in Sexual Activity (2)	53.0 (52.4, 53.6)	49.6 (48.2, 51.1)	49.5 (46.0, 53.0)	46.6 (45.8, 47.5)	46.2 (44.6, 47.7)	49.6 (47.8, 51.4)
Erectile Function (11)	50.5 (49.9, 51.1)	50.0 (48.6, 51.4)	50.2 (48.1, 52.2)	—	—	—
Vaginal Lubrication for Sexual Activity (6)	—	—	—	50.6 (49.8, 51.3)	54.2 (53.0, 55.4)	52.2 (51.0, 53.5)
Vaginal Discomfort with Sexual Activity (11)	—	—	—	49.3 (48.5, 50.0)	45.5 (44.2, 46.9)	49.1 (47.8, 50.4)
Vulvar Discomfort with Sexual Activity—Clitoral (4)	—	—	—	49.6 (49.1, 50.1)	50.8 (49.5, 52.2)	50.4 (49.4, 51.3)
Vulvar Discomfort with Sexual Activity—Labial (4)	—	—	—	49.4 (48.9, 50.0)	48.4 (47.5, 49.3)	51.7 (50.4, 52.9)
Anal Discomfort with Sexual Activity (6)	50.1 (49.1, 51.2)	59.4 (55.8, 63.0)	60.1 (49.9, 70.3)	49.8 (49.1, 50.6)	49.2 (48.5, 49.9)	53.1 (51.0, 55.3)
Oral Discomfort with Sexual Activity (6)	50.8 (49.8, 51.8)	55.6 (52.7, 58.6)	55.6 (50.5, 60.8)	49.8 (48.9, 50.6)	53.5 (50.3, 56.7)	54.9 (53.0, 56.8)
Oral Dryness with Sexual Activity (3)	50.2 (49.4, 51.0)	54.4 (52.3, 56.5)	55.4 (52.1, 58.7)	50.0 (49.3, 50.7)	54.5 (51.9, 57.0)	56.9 (54.3, 59.6)
Orgasm—Pleasure (3)	51.1 (50.5, 51.7)	48.9 (47.6, 50.2)	46.7 (44.0, 49.5)	48.8 (48.0, 49.6)	47.9 (45.9, 49.9)	49.4 (47.8, 51.0)
Orgasm—Ability (1)	52.0 (51.4, 52.6)	52.2 (50.8, 53.7)	50.6 (48.2, 53.1)	46.7 (45.7, 47.6)	50.3 (48.2, 52.4)	48.0 (46.2, 49.7)
Satisfaction with Sex Life (5)	51.2 (50.6, 51.8)	46.8 (45.6, 48.0)	46.0 (43.5, 48.5)	49.4 (48.6, 50.2)	47.4 (45.8, 49.1)	49.4 (47.8, 51.0)

^a^ PROMIS Sexual Function and Satisfaction scores for US adults who reported any sexual activity (with or without a partner) in the past 30 days. The US mean score is 50 with SD of 10.

## Discussion

Using data from two large, probability-based sample surveys of US adults, multiple domains of sexual function and satisfaction differed by sexual orientation, with differences ranging from 1/4 to nearly 1 standard deviation—sizes likely to be clinically meaningful. In contrast to a previous study that found more erectile difficulties reported by gay men compared to heterosexual men [5], we found very similar average erectile function scores for heterosexual, gay, and bisexual men. We did not find differences in orgasm-ability among these groups either, yet both gay and bisexual men reported lower sexual satisfaction compared to heterosexual men. Among women, we did not find differences in satisfaction by sexual orientation, in contrast to a previous study that compared heterosexual and lesbian women [6]. However, we did find differences in the domains of interest, lubrication, vaginal discomfort, and orgasm-ability. Notably, for sexual minority men and women, decrements were observed in domains of sexual function that have not traditionally been included in multi-dimensional self-report measures, including labial discomfort, anal discomfort, and oral discomfort and dryness. Sexual activity status and relationship status also differed by sexual orientation and may be related to the differences in function and satisfaction that we observed. These and other potential reasons for differences should be studied in future research.

Our study was limited in that, as with all sample surveys, respondents may have differed from nonrespondents in important ways. Due in part to our focus on meeting a target sample size rather than a specific response rate, our response rates did not reach 60%. Moreover, some may have declined to participate because of the topic. We selected GfK’s KnowledgePanel because of its probabilistic recruitment methodology based on mailing addresses and their multi-staged techniques for weighting to optimize representativeness to the English-speaking, US adult population. Our study also has particular strengths. Our sample sizes were large overall, allowing us to compare across the 6 groups. We also used one of the most robust measures of sexual function and satisfaction available to limit measurement bias.

There have been calls for clinicians to have greater awareness of their patients’ sexual concerns and dysfunction [16, 17]. Our data suggest that clinicians should also be aware that sexual minority patients are more vulnerable to certain sexual difficulties than heterosexual patients. Assessing sexual function in the context of clinical care can be challenging for a variety of reasons. Future research should examine whether clinical use of a comprehensive measure, such as the PROMIS SexFS v2, could promote efficient and sensitive identification of problems.
